# Enhanced Antitumor Efficacy and Reduced Cardiotoxicity of Ultrasound-Mediated Doxorubicin Delivery by Microbubble-Liposome Complexes

**DOI:** 10.1016/j.ultrasmedbio.2025.04.010

**Published:** 2025-05-13

**Authors:** Mingyu He, Xucai Chen, Francois Yu, Bin Qin, Huizhu Wang, Linda Lavery, Flordeliza S. Villanueva

**Affiliations:** Center for Ultrasound Molecular Imaging and Therapeutics, Pittsburgh Heart, Lung, Blood and Vascular Medicine Institute, University of Pittsburgh, Pittsburgh, PA, USA

**Keywords:** Ultrasound, Polymer microbubble, Liposomal doxorubicin, Soft tissue sarcoma, Cardiac function, Myocardial fibrosis

## Abstract

**Objective:**

Doxorubicin (Dox) is standard of care for treatment of sarcomas, but cumulative dosing is often limited by cardiotoxicity. We hypothesized that ultrasound targeted microbubble (MB) cavitation (UTMC) of a liposomal doxorubicin (LDox) conjugated polymer microbubble complex (DoxLPX) would enhance tumor inhibition and limit Dox cardiotoxicity.

**Methods:**

DoxLPX was intravenously injected in MCA205 sarcoma-bearing mice and concurrent ultrasound was delivered to the tumor site (DoxLPX + UTMC). Other mice received equivalent dosages of free Dox, LDox, or LDox + MB co-administration with UTMC (LDox + MB + UTMC). Tumor size and cardiac function were serially imaged with ultrasound. Postmortem cardiac tissue was analyzed for apoptosis. Biodistribution of Dox was performed with bioluminescence imaging postmortem where Cy5.5 was used as a fluorescent Dox analog.

**Results:**

DoxLPX + UTMC showed increased drug concentration in the tumor, a significant slowdown in tumor growth and prolonged median survival time. LDox and DoxLPX formulations had reduced drug extravasation into the myocardium. LDox + MB + UTMC also demonstrated superior tumor growth inhibition compared to free Dox and LDox. Three weeks after treatment commenced, DoxLPX + UTMC group showed significantly better left ventricular function indices than the free Dox group, consistent with biodistribution findings. Concordantly, heart tissue showed normal architecture of cardiac myocytes and significantly less interstitial/perivascular fibrosis in the DoxLPX + UTMC group.

**Conclusions:**

DoxLPX formulation in conjunction with ultrasound provides a targeted drug delivery platform with superior anti-tumor efficacy and reduced cardiac toxicity compared with systemic administration of free Dox.

## Introduction

Doxorubicin (**Dox**) is an anthracycline and is one of the best-understood and most widely used chemotherapeutic agents. However, Dox induces cardiac damage in an irreversible and cumulative dose-dependent manner. Dox dosing is largely determined by the maximal tolerated cumulative dose (400–550 mg/m^2^) [1]. A growing number of cancer survivors are at increased lifetime risk of Dox-induced cardiotoxicity. The onset of cardiotoxicity may be delayed as many as 10–15 years after the cessation of chemotherapy. The use of combination regimens with overlapping toxicities is likely to potentiate Dox-induced cardiotoxicity (e.g. cyclophosphamide, trastuzumab, taxanes) [2].

Pegylated liposomal Dox (**PLD**) (e.g. Doxil^®^, Caelyx) and non-pegylated liposomal Dox (LDox) (e.g. Myocet, D-99) formulations are alternative formulations of Dox designed to have reduced cardiotoxicity and improved pharmacokinetic profiles [3]. Doxil and Caelyx are FDA-approved treatments of AIDS-related Kaposi’s sarcoma and ovarian cancer [4]. Intravenously injected LDox formulations should not escape the vascular space in organs that have tight capillary junctions, such as the myocardium and gastrointestinal tract, while drug delivery to tumor sites with leaky vasculature is enhanced. PLD has a polyethylene glycol (**PEG**) coating around the liposome bilayer to protect the molecule from phagocytosis. However, PLD did not improve the maximally tolerated dose compared to free Dox and has a preferential concentration in the skin and drug leakage from capillaries in the palms of the hands and soles of the feet, causing another dose-limiting toxicity syndrome called palmar-plantar erythrodysesthesia (**PPE**) [5]. Thus, there remains a need to develop better targeted strategies for tumor delivery of Dox.

Dox is the standard of care for treatment of soft tissue sarcomas (**STS**), which are malignant tumors that originate from extra-skeletal connective tissues and that can arise at any site. Tumor response correlates with the Dox dosage, making Dox toxicity-related dose limitations particularly problematic for efficacy of therapy [6]. LDox was found only to be as effective or non-inferior to free Dox in treatment of STS [6,7]. The therapeutic efficacy and adverse side-effects of Dox – both dose-elated – in addition to the known benefits of regional therapies, define a need for a local non-invasive Dox delivery platform with low systemic toxicity for the treatment of STS.

Microbubbles (**MBs**) are ultrasound contrast agents comprising intravenously injectable gas-filled microspheres encapsulated by a biocompatible shell (lipids, proteins, or polymers). Ultrasound-targeted MB cavitation (**UTMC**), the alternating compression and expansion of MBs under the influence of an ultrasonic field, is an approach for image-guided local delivery of drugs or oligonucleotides that enables triggered unloading of cargo at the region of interest [8,9]. UTMC as a theranostic drug delivery strategy has various advantages, including its ability to reach deep tissue in a noninvasive manner and local regional application, while enabling concurrent ultrasound imaging to guide treatment location. UTMC causes transient hyperpermeability of vascular walls and the cell membrane, facilitating extravasation of large impermeant molecules into the extravascular space and/or endocytosis-independent uptake of payloads into cells (through sonoporation) [10].

UTMC combined with nanoscale LDox is a promising drug delivery strategy: As the LDox drug effect largely depends on its penetration through vascular endothelium and tumor interstitium, the ability of UTMC to increase endothelial barrier permeability should facilitate LDox efficacy. Indeed, UTMC has been shown to enhance the accumulation and permeation of the liposomal drug in tumors [11]. Lipopolycomplex (**DoxLPX**), a form of LDox conjugated to MBs, was designed as an advanced delivery vehicle to achieve non-invasive, spatially controlled targeted delivery by UTMC [8,12–15]. In *in vitro* studies, DoxLPX and ultrasound (US) treatment killed cancer cells by the local release of free Dox as well as enhancement of the cellular drug uptake via sonoporation [4,8]. In our prior *in vitro* work, DoxLPX + UTMC was superior in killing SCCVII murine squamous cell carcinoma cells compared to co-injection of LDox and MB + UTMC, possibly because proximity of LDox attached to vibrating MBs (DoxLPX) further enhanced liposomal Dox release and placed the liposomes more closely to areas of UTMC-induced sonoporation [8].

Another potential advantage of DoxLPX over LDox + MB coinjection + UTMC for *in vivo* use is that co-injection of LDox and MB does not preclude LDox extravasation and accumulation in non-target tissues, e.g. skin capillaries, whereas DoxLPX does not extravasate due to its size (~3 microns in diameter). Whereas prior studies have reported liposomal-MB formulations to achieve US-triggered drug release and uptake, they have not provided *in vivo* data on antitumor efficacy, cardiac toxicity or drug biodistribution [4,8,16]. Accordingly, in this study, we systematically analyzed the local delivery and antitumor efficacy of DoxLPX, combined with therapeutic US, and the cardiac safety of this strategy.

## Methods

### Preparation of DOX formulations

#### Free Dox.

Dox (>98% purity) was purchased from Cayan Chemical (Ann Arbor, MI).

#### Biotinylated Dox-loaded liposomes (liposomal Dox, LDox).

Biotinylated liposomes were prepared and loaded with Dox as described in Supplemental methods.

#### Polymer microbubbles.

Polymer MBs (3.3 *μ*m average diameter) were composed of an outer layer of crosslinked human albumin, an inner shell of (poly-D,L-lactide, PLA) and a core of nitrogen gas. Methods for synthesizing polymer MBs are detailed in Supplemental methods.

#### Liposomal Dox-loaded polymer microbubble complexes (DoxLPX) and FITC-lipoplexes (FITC-LPX).

**Figure 1.**
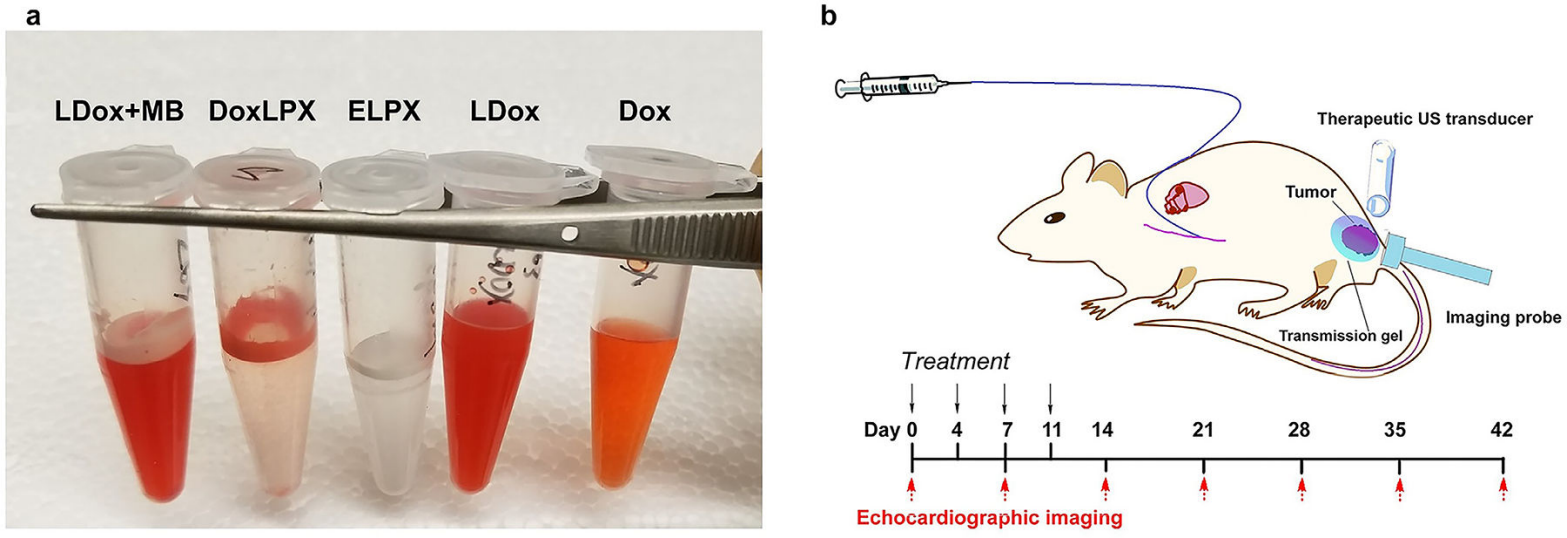
*In vivo* mouse tumor treatments. (a) Dox-containing formulations used in the tumor growth inhibition study: in each treatment 1.7 × 10^8^ LDox + MB, DoxLPX, or LDox (equivalent dose of 100 μg Dox), or 100 μg free Dox was injected. ELPX (1.7 × 10^8^) and saline were used as controls; (b) Schematic diagram depicting the intravenous infusion through the jugular vein and concurrent UTMC treatments (4 times) during infusion. Echocardiographic imaging was performed every 7 days.

Our Dox-loaded MBs (Doxloaded lipoplexes or **DoxLPX**) were synthesized by conjugating biotinylated polymer MBs to biotinylated LDox using biotin-avidin linking chemistry, as described in Supplemental methods (Fig. 1a) [8]. A negative control MB formulation comprised empty liposomes (no Dox) conjugated to polymer microbubbles (empty lipoplexes, **ELPX**).

### In vitro studies of ultrasound-induced Dox release from DoxLPX

To confirm release of Dox in response to ultrasound and to identify optimal acoustic parameters conferring maximal release in a short period of time (due to relatively rapid transit of DoxLPX through the tumor), we used a previously described *in vitro* experimental setup employing a degassed deionized water tank maintained at 37°C and equipped with a calibrated transducer [8]. Treatment ultrasound was provided by a 1 MHz flat single element transducer (A302S, 25.4 mm in diameter Olympus NDT, Waltham, MA) immersed in the tank and excited with an arbitrary function generator (AFG3252, Tektronix, Beaverton, OR, USA) and a gated radio frequency power amplifier (250A250AM8, Amplifier Research, Souderton, PA, USA). The ultrasound field was calibrated with a 200-μm capsule hydrophone (HGL-0200, Onda Corp, Sunnyvale, CA, USA).

In our prior *in vitro* work, we found that long ultrasound duty cycles at low acoustic pressure resulted in maximal Dox release from DoxLPX over 5 minutes in a static culture dish [8]. We posited that microstreaming, resulting from MBs stably oscillating in response to long pulses, facilitated drug release from the attached dox-containing liposomes; but maximal release took about 5–6 minutes to occur. In contrast, unlike the static cell culture system, in an *in vivo* situation, blood flow carries LDox or DoxLPX quickly through the tumor, and the *rate* of drug release within the tumor in response to ultrasound becomes relevant; i.e. the drug construct should release as much drug as possible during its brief transit through the tumor. We therefore needed to define an ultrasound regime that rapidly releases cytotoxic drug levels for *in vivo* use. Accordingly, we chose to test three ultrasound protocols (Pulses 1, 2, and 3, respectively, see Table 1) that would approximate varying cavitation regimes: A low acoustic pressure at 170 kPa (stable cavitation); a high acoustic pressure at 1 MPa (inertial cavitation); and a complex waveform combining a high acoustic pressure followed by a low acoustic pressure (combination of inertial and stable cavitation). The spatial peak temporal average intensities (*I*_SPTA_) were 0.48, 0.33, and 0.80 W/cm^2^, respectively. DoxLPX concentration was adjusted to 4.7 × 10^6^ MB/mL in PBS with a loaded Dox concentration of 2.63 μg/mL. The acoustic attenuation was relatively low at this concentration of DoxLPX for the ultrasound parameters used. For each experiment, a 500 μL volume of DoxLPX suspended in PBS was gently magnetically stirred in a polystyrene tube positioned at 40 mm from the transducer surface.

### In vivo studies comparing effects of varying Dox formulations on tumor growth and cardiac function

#### Murine sarcoma model.

The animal protocols were approved by the University of Pittsburgh Institutional Animal Care and Use Committee and conform to the Policy on Human Care and Use of Laboratory Animals. Eight-week-old female C57BL/6 mice were obtained from Jackson lab and housed in the animal facility of the Division of Laboratory Animal Resources at the University of Pittsburgh. Female mice were used due to their greater susceptibility to Dox cardiotoxicity [17,18]. At 9 weeks of age, mice were inoculated subcutaneously with 1.5 × 10^6^ MCA205 murine sarcoma cells (generously provided by Dr. Walter J. Storkus at the University of Pittsburgh School of Medicine) in the lower flank near the base of the tail and tumors were allowed to grow prior to treatment.

#### Therapeutic ultrasound.

UTMC was delivered with a single-element immersion transducer (A303S, 12.7 mm in diameter, Olympus NDT), driven by an arbitrary function generator (AFG3252, Tektronix) connected to a gated radio frequency power amplifier (Model 250AM8, Amplifier Research). The ultrasound field was calibrated with a 200-μm capsule hydrophone (HGL-0200, Onda Corp). Based on *in vitro* drug release studies described below, Pulse 3 combining high and low pressure pulses (Table 1) was used for all *in vivo* studies. The pulse was repeated 500 times, with the pulse train repeated every 2.5 s to allow MB reperfusion into the target area. The overall duty cycle was 10%. The spatial peak temporal average intensity was 0.16 W/cm^2^. The ultrasound transducer was positioned toward the tumor, tilted toward the tail of the mouse to exclude ultrasound exposure of the abdominal area and the heart. UTMC was continued for 3–5 minutes after the i.v. injection to achieve complete MB destruction (12–14 min total sonication time).

#### Serial ultrasound measurement of tumor volume.

Tumor growth was evaluated by high-resolution 3D ultrasound imaging of the tumor using automated scanning (Vevo 2100, VisualSonics, Ontario, Canada) at 21 MHz with a 0.2-mm step size between cross-sections. Tumor cross-sections were manually traced, and tumor volumes were calculated from 3D reconstructions of the outlined area. This technique can measure subcutaneous tumors with an irregular shape with an accuracy of ± 20 mm^3^. Tumor growth over time was fit to an exponential curve $(y)=X_{0}\cdot e^{kt}$, where $X_{0}$ represents initial tumor volume, and t stands for time in days. Doubling time (**DT**) were calculated as DT=(Ln2)/k.

#### Serial echocardiography.

To evaluate relative effects of differing Dox treatments on cardiac function, serial echocardiography was performed. Mice were lightly anesthetized with 0.9~1.2% isoflurane until the heart rate stabilized at 400 to 500 beats per minute. Two-dimensional short-axis images were obtained using a high-resolution echocardiography (Vevo 2100, VisualSonics) in B-mode and M-mode from the parasternal short-axis view. Left ventricular ejection fraction (**EF**), fractional shortening (**FS**), left ventricle (**LV**) internal end-diastolic diameter (**LVIDd**), LV internal end-systolic diameter (**LVIDs**), LV posterior wall diastolic thickness (**PWd**), LV posterior wall systolic thickness (**PWs**), and LV mass were measured or calculated using the Vevo LAB software (version 1.7.1, VisualSonics) in M-mode. LV mass index was calculated as LV mass/mouse body weight (g) on the day of measurement.

#### Protocol for *in vivo* treatment of MCA 205 tumor-bearing mice.

Treatments with Dox formulations commenced when tumor volume reached 40–90 mm^3^. Six groups of mice were studied as follows (*n* = 8–9 mice per group): Dox-treated mice received 100 μg DOX as: (1) free Dox; (2) LDox; (3) DoxLPX + UTMC; or (4) co-injection of LDox + MB + UTMC; and control mice received (5) ELPX (empty liposomes conjugated to MBs) + UTMC; or (6) saline. On the first day of treatment (Day 0), the tumor-bearing mice were anesthetized with 2% isoflurane in oxygen and an indwelling catheter was placed in a central vein to facilitate multiple intravenous infusions over time (Fig. 1b). The therapies (total volume 380 *μ*L) were intravenously infused in the jugular vein at 2.5 mL/h, and given on Days 0, 4, 7, and 11 (total 4 treatments; 20 mg/kg cumulative Dox dosage, excluding the saline and ELPX + UTMC controls). Rarely, the catheter became occluded by the third or fourth treatment, requiring tail vein injection as an alternative route for drug infusion.

Tumor volume and cardiac function were serially evaluated for up to 42 days using ultrasound imaging. Echocardiography was performed every 7 days starting from Day 0, the first treatment. On Day 42, or if tumor volume exceeded 2,000 mm^3^ before Day 42, the mice were euthanized under deep anesthesia (5% isoflurane). The tumor and heart were harvested, washed with cold saline, and cryopreserved or fixed with 10% formalin overnight, embedded in paraffin block and sectioned for further histology analysis.

### In vivo biodistribution studies: Fate of Dox after in vivo delivery

Cyanine5.5-amine (Cy5.5-NH_2_, Lumiprobe, Hunt Valley, MD) was used as a surrogate for Dox based on its similarity in molecular structure, moderate water solubility, and strong near-infrared wavelength fluorescence. Liposomal Cy5.5-amine (LCy5.5) and Cy5.5-LPX were formulated using the same method used for LDox and DoxLPX preparation, respectively. Separate C57BL/6J mice bearing 100–150 mm^3^ MCA 205 sarcoma tumors were intravenously injected with 360 μL of free Cy5.5-NH_2_, LCy5.5, Cy5.5LPX or LCy5.5 + MB (co-injection) (equivalent 40 μg Cy5.5-NH_2_ per mouse) and treated with the same ultrasound regime as for the tumor suppression studies (*n* = 3 mice per group). At 3.5 h after treatment, 350–500 μL blood was collected from the orbital sinus, the mice were euthanized under deep anesthesia, and the organs were harvested. Specimens of blood, tumor, spleen, kidney, liver, heart, lung, brain, skin on the back, muscle, bone (femur), and foot were imaged using a Xenogen IVIS 200 imaging system [19]. and further quantified using lysed tissue samples using a plate reader (see Supplemental methods).

### Tumor and cardiac tissue analysis

Sirius red/fast green staining was performed on heart tissue sections post-fixed in paraffin blocks [20]. Images were obtained on an Olympus IX81 microscope interfaced with a digital CCD camera (Olympus DP71) and quantitated using ImageJ 1.5 software. Caspase-3 activity assay was performed to assess for apoptosis in tumor tissue (see Supplemental methods).

### Statistical methods

All data are expressed as mean ± SD. Tumor volume and DT were compared using one-way ANOVA, with *post hoc* testing using Tukey post hoc (Prism 7.0, GraphPad). EF, FS and left ventricular (mass index data within each treatment group were compared using One-way ANOVA followed by a two-tailed t-test using Tukey post hoc (Prism 7.0, GraphPad). The percentage of mice with changes in EF, FS and LV mass index, categorized as mild (ΔEF<11%, ΔFS<20%, or ΔLV mass index<20%); moderate (11%≤ΔEF≤15%, 20%≤ΔFS≤30%, or 20%≤ΔLVmass≤40% mass index); or severe (ΔEF>15%, ΔFS>30%, or ΔLV mass index>40%), was compared using Chi-square test, and differences between two groups were compared using Fisher’s exact test. Kaplan-Meier survival curves were compared between groups using the log-rank test for trend.

## Results

### Characterization of LDox and DoxLPX

**Figure 2.**
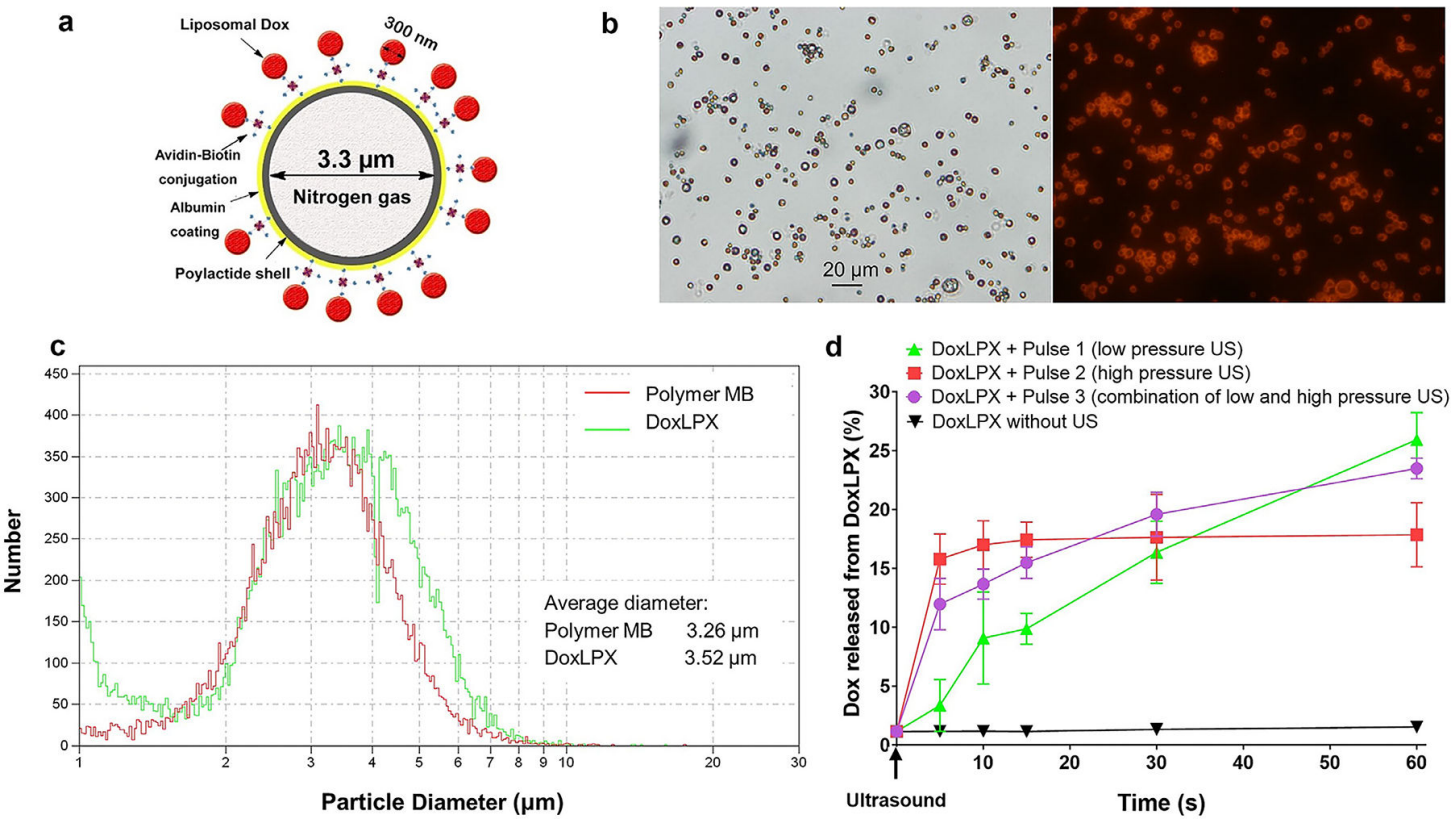
Characterization of DoxLPX. (a) Schematic structure of DoxLPX; (b) Brightfield and fluorescence microscopic images of DoxLPX (40 ×); (c) Size distribution of MB and DoxLPX; (d) *In vitro* Dox release kinetics from DoxLPX exposed to 3 different US pulses *in vitro*.

A schematic structure of DoxLPX is shown in Figure 2a. The LDox had a z-average size of 307.6 nm and a polydispersity index of 0.106 as measured by the Zetasizer. After conjugation of LDox to the polymer MB, the resulting DoxLPX demonstrated red fluorescence (Fig. 2b) and a slightly larger diameter compared to the polymer MB (3.52 ± 1.17 *μ*m vs. 3.26 ± 1.05 *μ*m, Figure 2c), consistent with Dox loading on the MB surface (Fig. 2b). By fluorescence dequenching assay using 0.3% Triton X-100, the magnitude of Dox loading on DoxLPX was 0.53–0.59 *μ*g Dox per 1 × 10^6^ DoxLPX.

Figure 2d shows the Dox release profile of DoxLPX over a 60 s period of exposure to each of the 3 experimental acoustic pulses (Table 1). DoxLPX did not release free Dox in the absence of ultrasound. The high-pressure ultrasound regime caused more rapid Dox release (17.0% release in 10 sec) compared to the low-pressure regime (9.1% release in 10 sec), but no further release beyond 10 seconds, likely due to MB destruction at high pressures. In contrast, the low-pressure pulse induced slow Dox release at 60 seconds, but continued Dox release from DoxLPX, amounting to 23.9% by 60 sec. The combination high and low pressure waveform achieved both a rapid and sustained release of Dox (13.7% at 10 sec, 24.9% at 60 s) and consequently was used in the remaining experiments.

### In vivo antitumor efficacy

**Figure 3.**
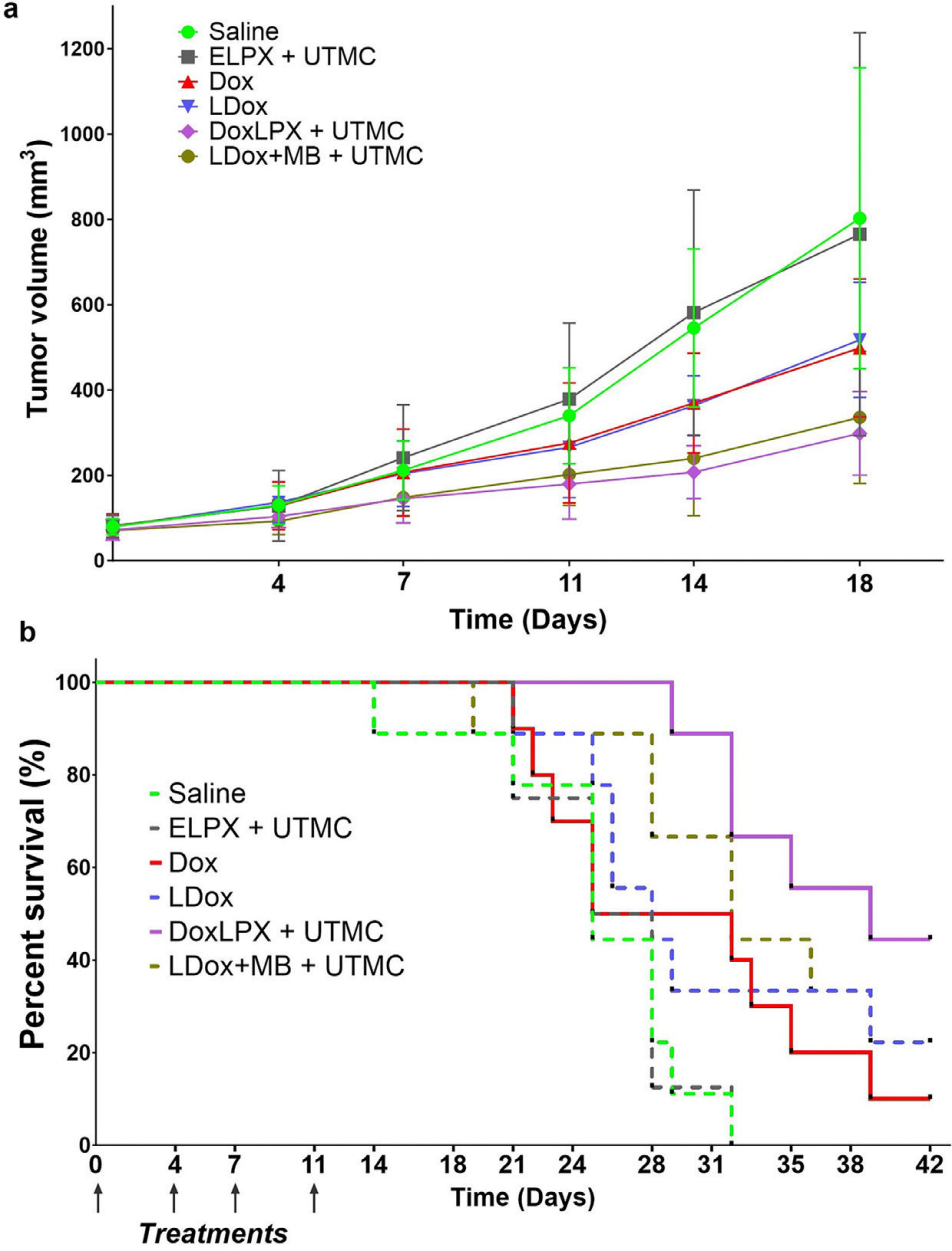
DoxLPX + UTMC has superior efficacy against MCA205 tumor. (a) Tumor growth plots (normalized to tumor size on Day 0). (b) Kaplan-Meier survival curves. *n* = 8–9 mice per group. See also Table 2.

Figure 3a shows tumor volumes up to 18 days, after which some mice required euthanasia due to tumor sizes exceeding 2,000 mm^3^. Negative control mice receiving either saline alone or ELPX + UTMC had rapid exponential tumor growth to 1008% and 941% of original tumor volume by day 18, respectively. Treatment with DoxLPX + UTMC or LDox + MB co-injection + UTMC delayed tumor growth the most, whereas treatment with free Dox or LDox conferred an intermediate growth inhibition by Day 18. In the 4 groups receiving any Dox formulation (100 *μ*g equivalent doses of dox), growth began to accelerate at a rate similar to that of the negative controls after day 14 (3 days after the last treatment). Mice receiving any of the 4 dox-containing formulations had smaller tumors compared to negative control mice receiving saline by Days 14 and 18 (*p* < 0.05), and ELPX + UTMC at Day 14 (*p* < 0.05) but not at Day 18 (*p* = 0.15) due to the large variability in the ELPX + UMTC group. While the mean size of tumors after treatment with DoxLPX + UTMC was not statistically different from tumors treated with LDox + MB co-injection (*p* = 0.5), it was only the DoxLPX + UTMC treated tumors that were statistically smaller than the free Dox (*p* < 0.005) and LDox-treated tumors (*p* < 0.001) at Days 14 and 18, likely due to the high variability in tumor responsiveness to the co-injection regime.

Treatment-related difference in tumor growth resulted in significant differences in tumor doubling time between the experimental groups (Table 2). Tumor doubling time in mice treated with any regime containing Dox was significantly more prolonged compared to that in the control mice receiving saline or ELPX + UTMC. The longest tumor doubling time occurred in mice treated with DoxLPX + UTMC (9.2 ± 2.7 days) and this was significantly longer than the doubling time of tumors treated with free Dox alone (*p* < 0.05), and longer than LDox treated group (*p* = 0.06). The tumor doubling time in mice receiving LDox + MB co-injection + UTMC group was significantly longer than negative controls, but not significantly longer than that in mice receiving free dox or LDox.

Tumor volume exceeding 2000 mm^3^ in size required mice to be euthanized for humanitarian reasons; thus, differences in tumor growth between groups resulted in differences in survival. As shown in the Kaplan-Meier survival curves (Fig. 3b), there was rapid fall-off in survival in the saline and ELPX + UTMC groups and by day 28, nearly 90% were dead, whereas all mice were still alive in the DoxLPX + UTMC group at day 28. Overall median survival was significantly longer in the DoxLPX + UTMC treated mice (38.5 days) compared to mice receiving free Dox (28 days, *p* < 0.03), LDox (28 days, *p* = 0.11), saline (24.5 days, *p* < 0.001), and ELPX + UTMC (26.3 days *p* < 0.001) mice (Table 2). Due to inconsistent efficacy in the co-injection group, the median survival time of mice receiving LDox + MB + UTMC co-injection was numerically but not significantly longer compared to the free Dox, or LDox groups.

### Changes in cardiac function in response to Dox treatment

**Figure 4.**
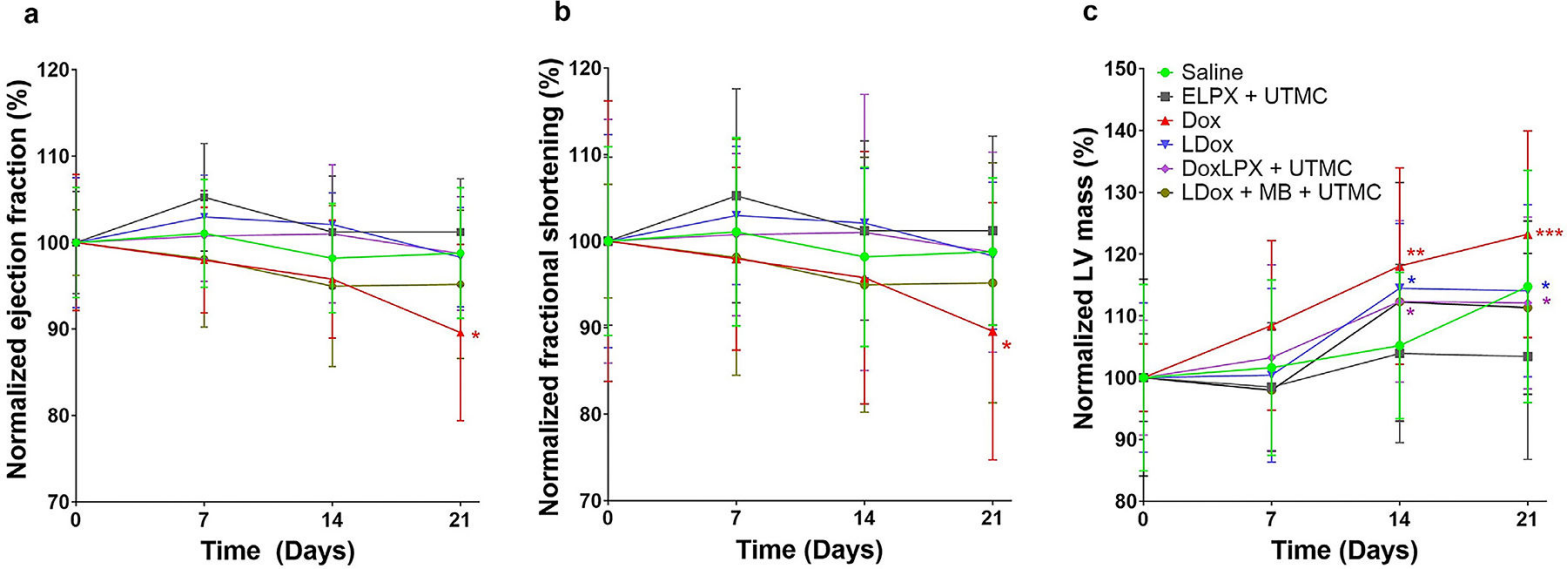
DoxLPX + UTMC preserves cardiac function. Serial echocardiographic data up to day 21, showing average (a) EF, ejection fraction; (b) FS, fractional shortening; and (c) LV mass index. Data are normalized to Day 0 values. *n* = 8–9 mice per group. *p < 0.05, **p < 0.01 vs. Day 0 data.

Figure 4 demonstrates serial echocardiographic measures of ejection fraction, fractional shortening and left ventricular mass. Echo measures were compared between and within groups up to day 21, after which mice with enlarging tumors exceeding 2,000 mm^3^ were euthanized. Negative control mice treated with saline or ELPX + UTMC had stable LV systolic function, fractional shortening, and LV mass during the study. Mice receiving free Dox had a progressive decline in ejection fraction and fractional shortening, such that by Day 21, these indices of systolic function were significantly less compared to baseline. Similarly, LV mass increased in the free Dox group, and was significantly greater than baseline by Day 14. In mice receiving co-injection of LDox and MB + UTMC, there was a trend towards a decrease in ejection fraction and fractional shortening, and an increase in LV mass by Day 14. Mice receiving LDox did not have a change in systolic function but had an increase in LV mass, which was significant at Day 14 compared to baseline. Importantly, mice treated with DoxLPX + UTMC had stable ejection fraction, fractional shortening, and less increase in LV mass during the study. These findings suggest that DoxLPX + UTMC uniquely spares the heart from Dox-related injury. Figure S1 plots the incidence of significant decreases in EF and FS, and the increases in LV mass as a function of treatment group. When comparing the 4 groups receiving any form of Dox, no mice receiving DoxLPX + UTMC experienced abnormal changes in these metrics, whereas the free Dox, LDox and LDox + MB co-injection + UTMC groups all included mice who developed abnormalities in these metrics, with the free Dox group having the most mice developing some form of cardiac abnormality (*p* < 0.05 vs. DoxLPX + UTMC for abnormal decrease in FS).

### Histological analysis of heart tissue

**Figure 5.**
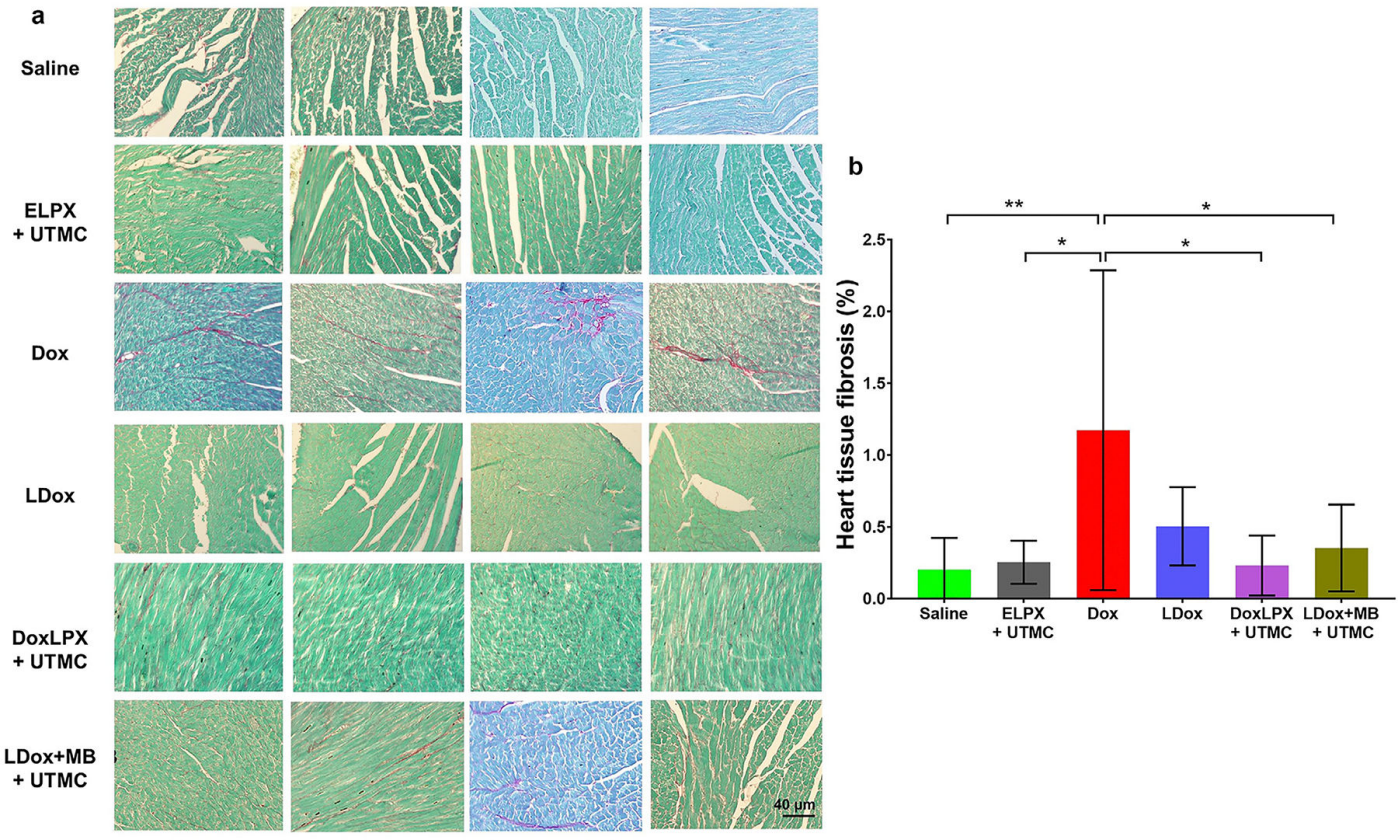
DoxLPX + UTMC protects against Dox-induced fibrosis. (a) Representative cardiac sections stained with Sirius red/fast green to detect interstitial fibrosis. Red: collagen fibrosis; Green: non-collagen components. (b) Quantification of fibrosis area in heart tissue by morphometry in ventricular cross-sections. *n* = 8–9 mice per group, data shown as mean ± SD.

Figure 5a demonstrates myocardial specimens after Sirius red/fast green staining for fibrosis. Free Dox administration induced moderate myocardial interstitial and perivascular fibrosis that was significantly greater than saline control or ELPX + UTMC (Fig. 5b). Compared to the free Dox group, there was significantly less myocardial fibrosis in mice receiving DoxLPX + UTMC. Mice receiving equivalent dosage of LDox had hearts with numerically less fibrosis compared to those receiving free Dox but the difference was not statistically significant (Fig. 5b).

### Caspase-3 activity in heart tissue

**Figure 6.**
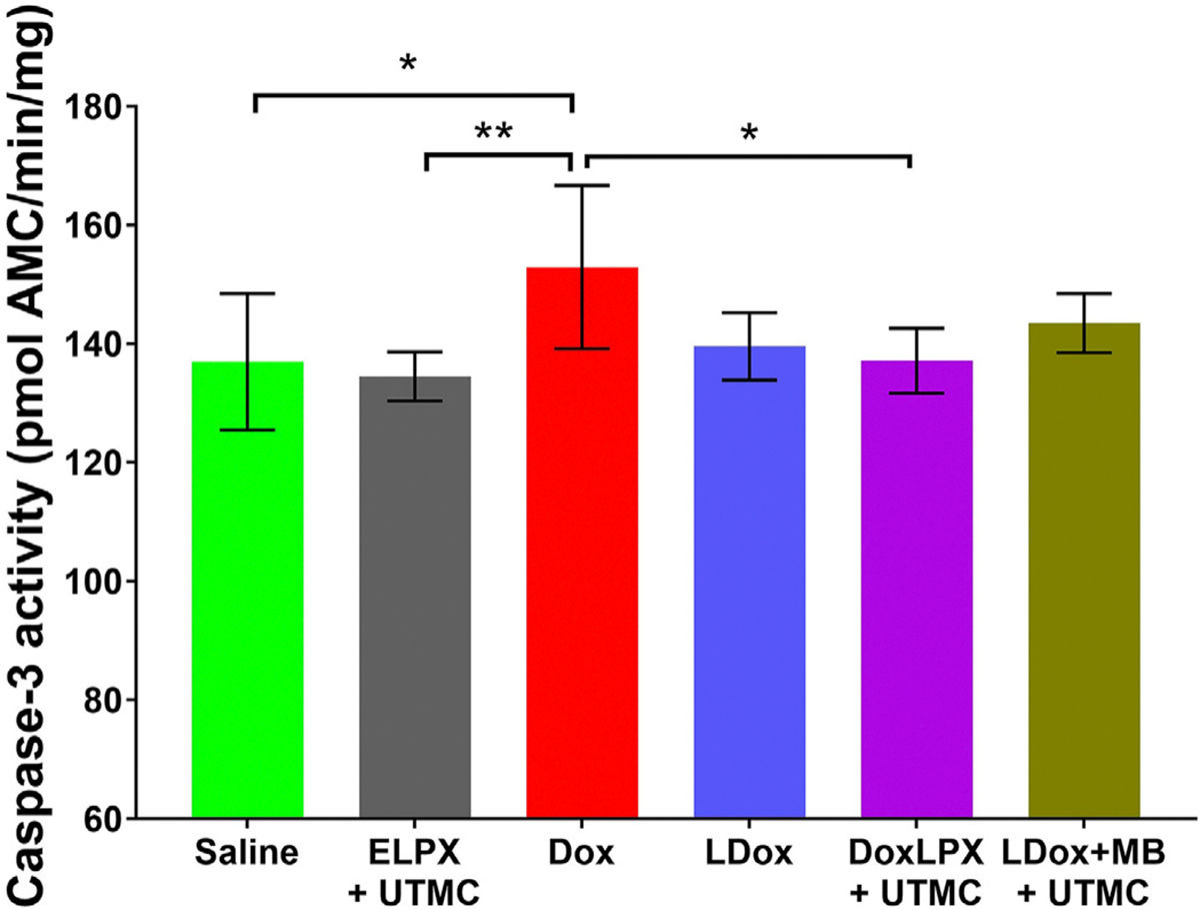
Caspase-3 activity in heart tissue homogenates. Free Dox caused more apoptosis (Caspase-3 activity) vs. other treatments. *n* = 8–9 mice per group, *p < 0.05, **p < 0.01.

Caspase activity, a marker of apoptosis, was quantified by fluorescence intensity of the substrate peptide for caspase-3. Administration of free Dox significantly increased myocardial caspase activity compared to saline treated or ELPX + UTMC treated mice (Fig. 6), whereas DoxLPX + UTMC attenuated free Dox-induced apoptosis, where myocardial caspase activity in Dox-LPX treated mice was less than that in free-Dox treated mice; indeed, myocardial caspase activity in DoxLPX + UTMC treated mice was no different from that in control mice receiving ELPX + UTMC or saline (Fig. 6). Cardiac caspase activity in mice receiving LDox or LDox + MB coinjection + UTMC was numerically lower but not statistically different than that in mice receiving free Dox.

### Biodistribution of drug delivery by LPX + UTMC in the tumor-bearing mice model

**Figure 7.**
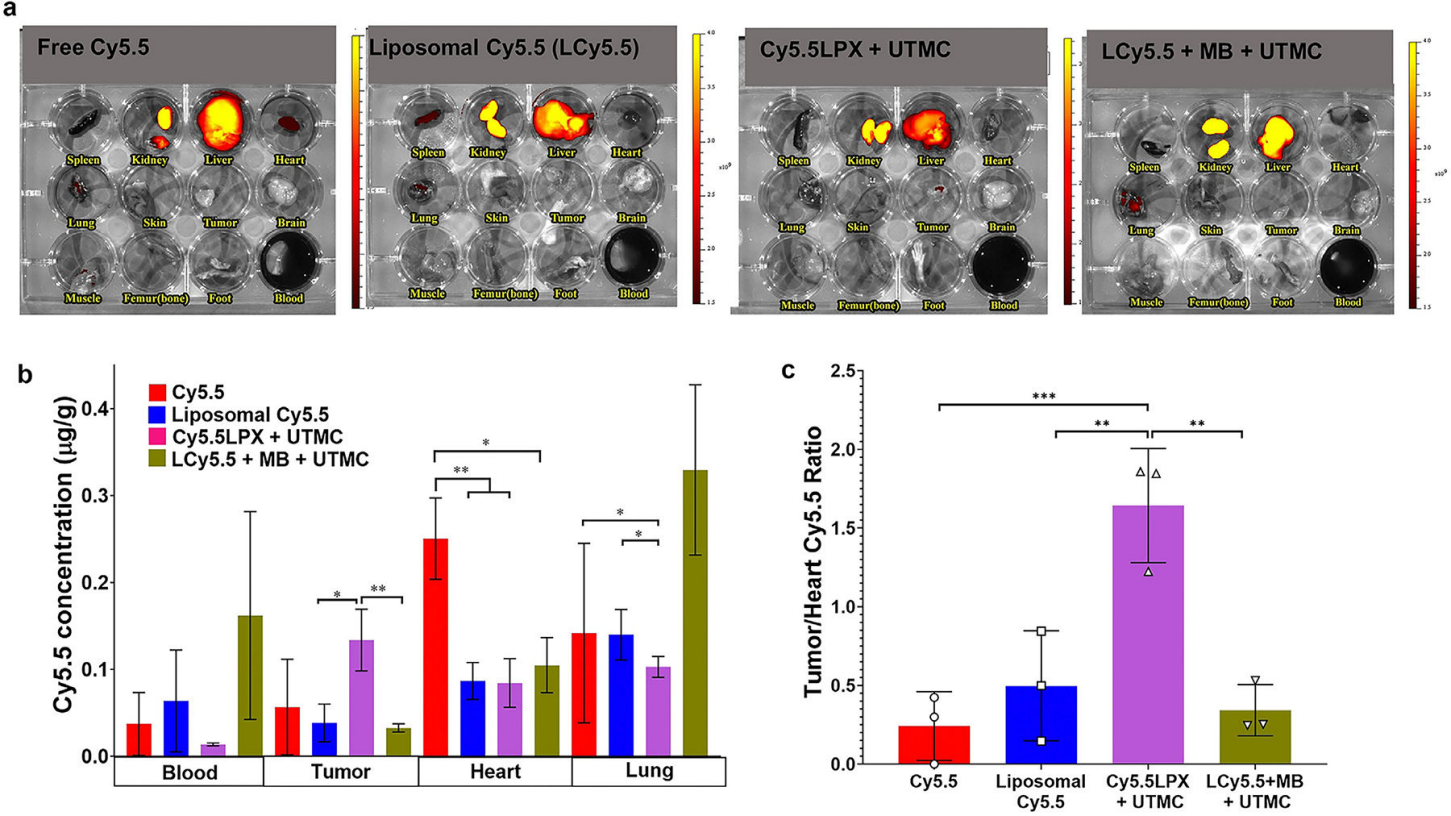
Representative *ex vivo* fluorescence images showing biodistribution of Dox analog (Cy5.5). (a) Representative *ex vivo* fluorescence images of organs excised 3.5 hrs after treatment, demonstrating higher intra-tumoral and lower cardiac uptake of Cy5.5 after Cy5.5LPX + UTMC (third panel from left); (b) Quantitative biodistribution (*n* = 3) of Cy5.5 concentration in tumor, blood, heart, and lung in tumor-bearing mice 3.5 h post *i.v.* injection of free Cy5.5, LCy5.5, LCy5.5 + MB + UTMC, and Cy5.5 LPX + UTMC; (c) Cy5.5 concentration ratio in tumor/heart tissue (*n* = 3 each group). *p < 0.05, **p < 0.01, ***p < 0.001.

Dox biodistribution resulting from the varying treatment regimens was assessed using post-mortem bioluminescence imaging of organs (*n* = 3) harvested 3.5 hours after treatment, using the fluorophore Cy5.5 as a surrogate for Dox in the free, liposomal, and the two lipoplex MB formulations (Cy5.5; LCy5.5; Cy5.5LPX + UTMC; or LCy5.5 + MB + UTMC, respectively). Figure 7a shows representative images for each group. Only the mice that received free Cy5.5 had noticeable Cy5.5 fluorescence in the heart and skeletal muscle. There was tumor uptake of Cy5.5 after Cy5.5LPX + UTMC that was not visualized in the other treatment groups. Quantification of Cy5.5 uptake was consistent with these qualitative findings (Fig. 7b). Cy5.5LPX + UTMC treatment significantly increased intratumoral Cy5.5 concentration (0.134 ± 0.036 μg/g) – by an order of magnitude – compared to LCy5.5 (0.038 ± 0.022 μg/g), LCy5.5 + MB + UTMC (0.033 ± 0.005 μg/g) and free Cy5.5 (0.057 ± 0.055 μg/g). There was significantly more Cy5.5 in the heart (0.250 ± 0.047 μg/g) after injection of free Cy5.5 compared to that after injection of LCy5.5 (0.087 ± 0.021 μg/g), Cy5.5LPX + UTMC (0.084 ± 0.028 μg/g) and LDox + MB + UTMC (0.105 ± 0.032 μg/g). As a result, the ratio of tumor to heart uptake of Cy5.5 was highest in the Cy5.5LPX + UTMC group (1.60 ± 0.32), and less than 1 in mice receiving free Cy5.5 (0.23 ± 0.22), LCy5.5 (0.50 ± 0.35), and LCy5.5 + MB + UTMC (0.34 ± 0.16) (Fig. 7c). Blood concentration of Cy5.5 in mice receiving free Cy5.5, LCy5.5 and LCy5.5 + MB + UTMC group was numerically higher than that of the mice receiving Cy5.5LPX + UTMC, but this was not statistically significant. Cy5.5 distribution in the other solid organs is shown in Figure S2.

## Discussion

Dox, a mainstay of chemotherapy for numerous cancers, is limited by cardiotoxicity, where effective tumor suppressive treatment must be discontinued due to dose-related left ventricular systolic dysfunction, or patients having undergone curative Dox treatment are left with ongoing or heightened risk for heart failure. The main finding of this study is that our novel DoxLPX delivery vehicle and UTMC delivery platform achieved preferential Dox delivery to sarcoma tumor while sparing the heart, resulting in superior tumor growth inhibition and preserved cardiac function compared to current clinically available Dox formulations (free dox, liposomal dox). The development of a targeted delivery platform that concentrates Dox in the tumor while sparing the heart could have major implications for cancer treatment.

Our MBs comprise a 3-*μ*m diameter nitrogen gas core stabilized by a biocompatible and biodegradable PLA shell. Polymer MBs are stabler in the circulation than more conventionally used lipid MB(14) after i.v. administration *in vivo*, reducing unwanted MB destruction or drug release in non-target regions unexposed to ultrasound. In our formulation, human serum albumin was crosslinked and coated on the PLA shell to minimize immunogenicity and to provide reactive thiol groups to conjugate liposomal drug during clinical application. Other groups have attempted to load MB formulations with Dox(4, 8, 12, 16); to best of our knowledge, our DoxLPX formulation has the highest Dox loading content.

We first determined the optimal ultrasound regime for timely and sustained release of Dox from DoxLPX. First, we showed that Dox release from DoxLPX was ultrasound-dependent, as there was no Dox release in the absence of ultrasound (Fig. 2d). Next, we sought to develop an optimal ultrasound regime for *in vivo* use. In our previous *in vitro* studies of DoxLPX + UTMC, we determined an acoustic regime that conferred the highest dox release in a static cell culture system, where sustained and maximal drug release could occur relatively slowly over the course of 5 minutes after UTMC [8]. However, in an *in vivo* system where blood flow carries DoxLPX more quickly through the tumor, we needed an acoustic regimen that would release Dox quickly in response to ultrasound (first 15 seconds, as MBs are transiting the microcirculation), while also allowing for more sustained release (defined here as 1 minute), as would occur from liposomes retained in tissue. Accordingly, we tested regimens involving low acoustic pressure, high acoustic pressure, and a combination of low and high acoustic pressures.

We had previously shown *in vitro* that low acoustic pressures, long cycle pulses were more effective than high pressures in releasing Dox from DoxLPX over the course of 1 minute [8,9]. In our previous study, we surmised that low acoustic pressures at long cycles agitated the surrounding medium and mechanically facilitated Dox release from attached liposomes, whereas high acoustic pressures immediately destroyed the MBs (inertial cavitation), allowing for some immediate, partial Dox release, but disallowing more prolonged mechanical agitation of the liposomes to achieve ongoing release with time. Similarly, in the current study, as shown in Figure 2d, we found that low acoustic pressure ultrasound caused more Dox release from DoxLPX by 60 s than the high acoustic pressure ultrasound (1 MPa). However, Dox release at low pressure was slow, with less than 10% release at 15 seconds, but it continued to increase, to nearly 25% by 1 minute. In comparison, Dox release after high pressure was higher – more than 15% at 15 seconds, but it plateaued thereafter, and was less than that after low pressure US at 1 minute (Fig 2d), likely because inertial cavitation destroyed many MBs (rapid early dox release), but there was no subsequent available mechanical agitation (by stable cavitation of MB) to facilitate dox release from residual liposomes. Therefore, to achieve a rapid and sustained release afforded by the high and low pressure regimes, respectively, we empirically customized a therapeutic US (pulse 3 in Fig. 2d), combining both high-pressure and low-pressure regimens. We surmised that inertial cavitation of the MBs from high acoustic pressure would cause acute and rapid disruption of liposomes (rapid dox release); then, the subsequent additional ultrasound pulses at lower acoustic pressure would allow stable cavitation of remaining MBs to mechanically agitate remaining liposomes to permit ongoing, slower dox release after the initial rapid release. Having found this combination pulse to confer both rapid and sustained Dox release, this pulse scheme was chosen for the *in vivo* experiments.

Our *in vivo* studies demonstrated the efficacy of DoxLPX + UTMC over other delivery strategies for suppressing tumor size and increasing survival (Fig. 3). Tumor doubling time was longer in all the Dox-treated groups compared to the saline-treated or ELPX + UTMC group. However, the Dox-LPX + UTMC mice had the longest mean doubling time among the Dox-treated mice (Table 2). The efficacy Dox or LDox treatment via i.v. injection showed survival benefit vs. the negative control mice on Kaplan Meir analysis; and the DoxLPX + UTMC mice had the longest median survival. Interestingly, the co-injection of LDox + MB + UTMC conferred a survival benefit as well vs. control mice, but not compared to free Dox-treated mice, likely due to greater variability of treatment response and survival in the co-injection group. Overall, not only was DoxLPX + UTMC better than negative control mice in prolonging tumor doubling time and survival, but it was also superior to free-Dox with respect to these metrics.

We found that cardiac performance and remodeling, echocardiographically measured in terms of ejection fraction, fractional shortening, and left ventricular mass, were best preserved in mice receiving DoxLPX + UTMC. Not unexpectedly, mice receiving free Dox at the known cardiotoxic dose administered in this study, had a deterioration in left ventricular ejection fraction and fractional shortening, and an increase in LV mass over the 21 day monitoring period. Mice receiving LDox and coinjection of LDox + MB + UTMC had a decrease in LV systolic function and increase in LV mass by Day 14, that was not as dramatic as that caused by free Dox, and which leveled off or improved by Day 21, perhaps reflecting some recovery after the last dose of Dox on Day 14. These findings suggest a relative cardioprotective effect associated with Dox delivery using DoxLPX + UTMC, which was also supported by tissue data: Histology indicated that free Dox caused significantly more myocardial fibrosis compared to negative control hearts. In contrast, myocardial fibrosis in mice treated with DoxLPX + UTMC was significantly less than that in mice treated with free Dox, and it was not different than negative control mice treated with saline or ELPX + UTMC. Consistent with this tissue finding, myocardial caspase activity indicative of apoptosis was highest in free Dox treated mice; compared to free Dox-treated mice, caspase activity was significantly less in mice treated with DoxLPX + UTMC, but not in mice treated with LDox or LDox + MB coinjection + UTMC.

The biodistribution data shed light on the mechanisms by which DoxLPX + UTMC caused superior tumor inhibition and improved survival, while at the same time being less cardiotoxic compared to the other Dox delivery strategies. Here, we used a fluorophore, Cy5.5, as an analog for Dox, and substituted it for Dox in the LPX, DoxLPX, LDox + MB and free Dox formulations. Cardiac accumulation of Cy5.5 was highest after free i.v. Cy5.5 injection, and significantly less after Cy5.5LPX + UTMC, Cy5.5LPX, or Liposomal Cy5.5 + MB + UTMC. Intratumoral concentration of Cy5.5 was highest, over double, after Cy5.5LPX + UTMC compared to that after Cy5.5 delivery using the other Dox formulations; thus, the tumor to heart concentration ratio was highest after DoxLPX + UTMC. These data indicate that DoxLPX + UTMC concentrates Dox within the tumor, via navigation of the ultrasound beam to the tumor. Further, because Dox release from DoxLPX is ultrasound-dependent, the heart, which is not within the ultrasound beam is spared from Dox release.

In addition to greater local tumor release of Dox from liposomes as a result of ultrasound induced MB cavitation, there are other potential mechanisms by which DoxLPX + UTMC treatment may induce greater intratumoral accumulation of Dox: After the high acoustic pressure component of the ultrasound regime, MB destruction may lead to fragments of liposomal “debris’ containing Dox, locally within the tumor. We and others have shown that UTMC may cause sonoporation and increased interendothelial gaps, leading to transient endothelial barrier hyperpermeability. The latter could permit extravasation of remnant liposomal particles across the vasculature, which could slowly release Dox over time. Acoustic cavitation induced endothelial hyperpermeability may also account for some of the therapeutic effect seen when MB are co-injected with LDox in the presence of US: transient hyperpermeability may have allowed LDox to leave the intravascular space into the tumor. Since the heart is not in the ultrasound field, these events would not occur in the coronary microcirculation; further, unlike LPX, which is sub-micron and can potentially leave the intravascular space, DoxLPX is micron sized, and remains exclusively intravascular in location, further providing another level of cardiac protection with DoxLPX.

Our study is consistent with the findings that DOX-loaded microbubbles and ultrasound increased the cellular uptake of Dox *in vitro* [4,8,21] and *in vivo* [15,22]. However, our study is distinct from previous studies in that we optimized our ultrasound parameters by using a combined low/high acoustic pressure that could be applied on live animals, assessed biodistribution to prove intra-tumoral accumulation of Dox by UTMC and importantly, concomitantly studied the cardiotoxicity difference of treated tumor-bearing mice receiving Dox, LDox or DoxLPX + UTMC. To our knowledge, this is the first time that targeted delivery and cardiotoxicity are measured in the same animals.

Several limitations in our study should be noted. We synthesized a Dox-loaded liposome, comparable to the commercially available Doxil; the liposome was biotinylated to allow its attachment to the microbubble using biotion-avidin bridging chemistry. This construct would not be clinically optimal due to immunogenicity of streptavidin; chemical conjugation of the liposomes to the microbubble using alternative chemistry would be another more clinically translatable alternative. Our study suggested potential clinical benefit from a more straightforward delivery strategy, namely coinjection of LDox + MB + UTMC, which could theoretically be clinically implemented since Doxil is on the market, and there are FDA approved MB formulations. This would not, however, eliminate the risks of subcutaneous accumulation of the liposomes nor the associated side effects.

## Conclusions

We developed a liposome-microbubble complex as an effective carrier of Dox for ultrasound-mediated tumor therapy. DoxLPX + UTMC treatment conferred significant survival benefit and tumor lowering effect compared to control (untreated or ELPX + UTMC) animals or mice receiving equivalent dose free Dox or liposomal dox. DoxLPX + UTMC had the highest intratumoral Dox concentration compared to other Dox delivery strategies, and the highest tumor to heart Dox accumulation ratios, accounting for the high antitumor efficacy with low cardiotoxicity. Tumor targeting is achieved by navigation of the ultrasound beam, resulting in Dox release either directly from the liposomes or due to UTMC-induced endothelial hyperpermeability to liposomal debris after US. DoxLPX + UTMC treated mice showed the least damage to the cardiomyocytes both histologically and by echocardiographic assessment of function and mass. These data suggest that DoxLPX + UTMC is a promising strategy for Dox delivery in cancer that addresses many of the current limitations of systemic Dox therapy.

## Supplementary Material

1

Supplementary material associated with this article can be found in the online version at doi:10.1016/j.ultrasmedbio.2025.04.010.

## Figures and Tables

**Table 1:** Ultrasound pulse configurations (1 MHz) for the *in vitro* study

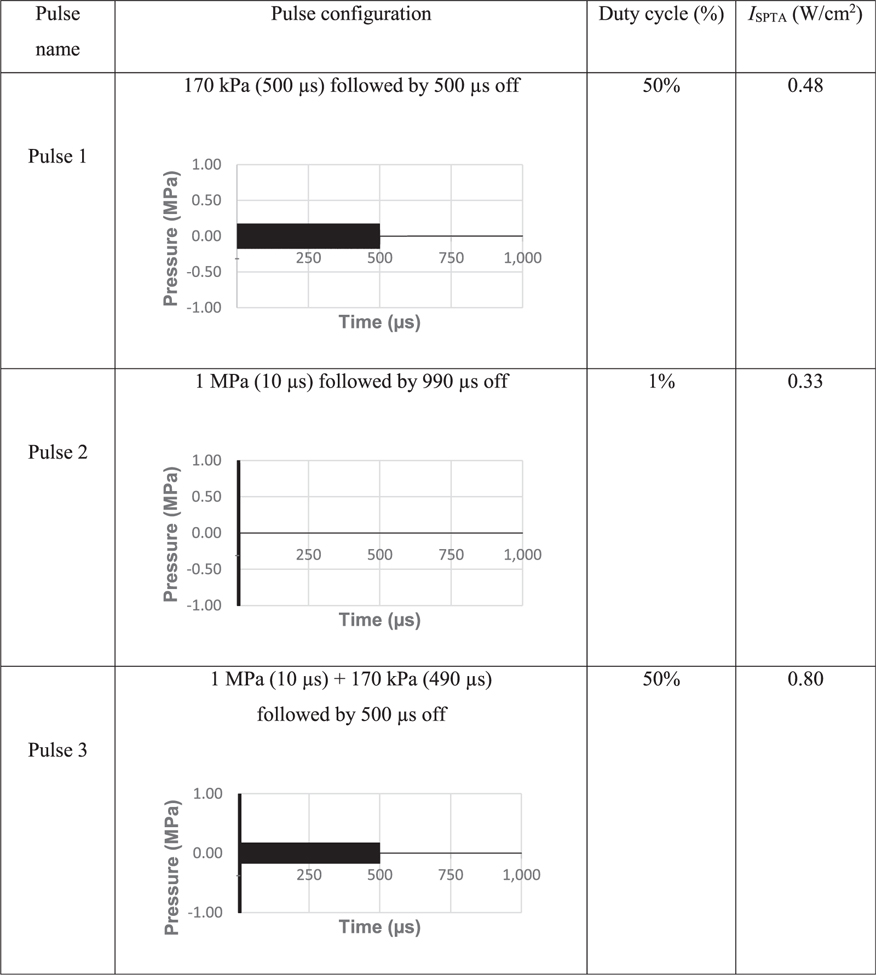

**Table 2:** Median survival time and mean tumor doubling time

	Saline	ELPX + UTMC	Dox	LDox	DoxLPX + UTMC	LDox + MB + UTMC

Median survival time (Days)	24.5	26.3	28.0	28.0	38.5^***,###,§^	31.5^**,##^
Mean Tumor doubling time (Day)	5.2 ± 0.5	5.7 ± 1.1	7.1 ± 1.0^**,#^	7.2 ± 0.7^***,##^	9.2 ± 2.7^**,##,§^	8.7 ± 2.5^**,##^

* *p* < 0.05,

** *p* < 0.01, and

*** *p* < 0.001 vs. saline group;

# *p* < 0.05,

## *p* < 0.01,and

### *p* < 0.001 vs. ELPX + US group;

§ *p* < 0.05 vs. Dox group.

## Data Availability

Data will be made available upon request.
