# Supplementary material for: Enhanced Antitumor Efficacy and Reduced Cardiotoxicity of Ultrasound-Mediated Doxorubicin Delivery by Microbubble-Liposome Complexes
Source: Ultrasound Med Biol. Author manuscript; Available in PMC 2026 Jun 9. (PMC13249505; doi:10.1016/j.ultrasmedbio.2025.04.010)
Supplement: 1 [file NIHMS2174833-supplement-1.pdf]

Supplementary methods

Preparation of biotinylated liposomes

L-α-phosphatidylcholine, hydrogenated (HSPC), cholesterol (Chol), 1,2-distearoyl-sn-glycero-3-phosphoethanolamine-N-[biotin (polyethylene glycol)-2000] (DSPE-PEG-Biotin) and 1,2-distearoyl-sn-glycero-3-phosphoethanolamine-N-[methoxy(polyethylene glycol)-2000] (ammonium salt) (DSPE-PEG) were purchased (>99% purity, Avanti Polar Lipids Inc, Alabster, AL) and mixed in 128:62:5:5 molar ratios. This formulation is similar to the Doxil® liposome, i.e. HSPC:Chol:DSPE-PEG in a molar ratio of 112:76:10.[18] All lipids were dissolved in dichloromethane (DCM) in a glass vial and then dried under a stream of argon gas. The lipid thin film was hydrated in a 300 mM ammonium sulfate solution at a 10 mg/mL lipid concentration. The lipid film was then gently sonicated briefly (Sonicator 75D, VWR, Radnor, PA) and frozen/thawed in five alternate cycles by immersing in liquid nitrogen and a 65ºC water bath. The resulting liposomes were then extruded through polycarbonate sizing filters (400 nm pore size, Whatman® Nuclepore Track-Etch Membranes, Buckinghamshire, UK). The liposomes were purified by passing through a Sephadex G-25 column (PD-10 column, GE Healthcare Bioscience, Pittsburgh, PA).

Dox-loading of liposomes

Dox loading was performed as follows: Dox solution (10 mg/mL) was added to the liposomes in a 9:25 weight ratio (Dox:lipid). The suspension was magnetically stirred and incubated at 65ºC for 1 h. Surplus (free) Dox was then removed by passing the suspension through Sephadex G-50 (20 mL gravity-flow chromatography column (Bio-Rad Laboratories, Hercules,

CA) and Sephadex G-75 (PD-10 column, GE Healthcare Bioscience). The mean hydrodynamic diameter of the final liposomes was determined by dynamic light scattering (Zetasizer Nano, Malvern Panalytical, Worcestershire, UK). The concentration of the incorporated Dox was measured by fluorometry at excitation/emission wavelengths of 485/595 nm (DTX-880 Multimode Detector, Beckman Coulter, Brea, CA) in 0.3% (v/v) Triton X-100 Tris-HCl buffer solution (50 mM, pH 7.0) to lyse the liposomes.

Preparation of polymer MBs

PLA was dissolved into isopropyl acetate and mixed with cyclooctane. The solution was slowly added into stirred medical grade human serum albumin (HSA) at 30˚C and emulsified by pumping through a stainless- steel filter (7 µm pore size) by a peristaltic pump for 15 min. Then the emulsion was added in 0.07% glutaraldehyde solution under vigorous stirring for 4 hours. HSA on the emulsion droplets was quickly crosslinked and the organic solvent was evaporated from the solution under mechanical stirring at 30˚C. The MB surface was biotinylated by reacting with EZ-link Maleimide-PEG2-Biotin (Thermo Scientific) according to the manufacture's instruction to convert the thiol group of human albumin to biotin. The result polymer MBs were washed 3 times by centrifuging the solution at 2,000 rpm for 10 min and the subnatant below the MB layer was discarded.

Preparation of Liposomal Dox-loaded polymer microbubble complexes (Dox-loaded lipoplexes, DoxLPX)

Briefly, 6×10^8 polymer MBs were incubated with 250 µL avidin (1.25 mg) in PBS at room temperature for 2 h, washed in PBS 3 times and centrifuged. Purified biotinylated LDox,

synthesized as above (1.5 mL) was mixed with the avidinated MBs at room temperature for 2 h, then centrifuged. Excess (unbound) liposomes in the subnatant were discarded, yielding a final product of MBs carrying Dox-loaded liposomes (Dox-lipoplexes, DoxLPX) floating in PBS (Fig. 1A and Fig. 2A). The DoxLPX were counted and sized by a Coulter counter (Multisizer 3, Beckman Coulter), and the amount of Dox loading was assessed by fluorometry using 0.3% Triton X-100 in Tris-HCl buffer solution (50 mM, pH 7.0). Conjugation of the liposome to the MB surface was verified by fluorescence microscopy (IX81, Olympus, Center Valley, PA) (Fig. 2B). A negative control microbubble formulation comprised empty liposomes (no Dox) conjugated to polymer microbubbles (empty lipoplexes, **ELPX**).

In vivo Dox biodistribution

To further quantify the distribution of Cy5.5-NH_2, tumor, spleen, kidney, liver, heart, and lung were wet- weighed and 50 μg of each tissue specimen were homogenized in 200 μL lysis buffer (50 mM HEPES, 50 mM NaCl, 1% Triton X-100, 5 mM EDTA, and 15 mM DTT) using a sonicator homogenizer (Sonicator Ultrasonic Processor XL, Misonix, Farmingdale, NY) in Eppendorf tubes immersed in an ice-water. The tissue samples were centrifuged at 12,000 RCF for 30 min and the blood samples were centrifuged bath at 1,500 RCF for 12 min at 4˚C. 50 μL supernatant of each specimen was added in black 384 well plates. Cy5.5-NH_2 fluorescence intensity of 3 parallel samples of each organ was determined at Ex/Em 680/710 nm using a plate reader (Infinite 200 PRO, Männedorf, Switzerland). Free Cy5.5-NH_2 in the lysis buffer in the range of 0~2 μg/mL was used to establish a standard curve.

Caspase-3 activity assay

Heart tissue was washed twice with cold PBS and lysed in lysis buffer (50 mM HEPES, pH 7.4, with 5 Mm CHAPS, and 5 mM DTT). Tissue lysates were centrifuged at 10,000 RCF for 15 min, and the total protein concentration was measured using a BCA assay kit (ThermoFisher Scientific, MA). Caspase-3 activity assay was performed in triplicate in 96-well plates. For each protease assay, 200 µL assay buffer (100 mM HEPES, pH 7.2, 10% sucrose, 0.1% CHAPS, 1 mM Na-EDTA, and 2 mM DTT) containing 50 µM Ac-DEVD-AMC (Enzo Life Sciences, Farmingdale, NY) was incubated with 50 µg lysate protein at 37˚C for 1 h. Caspase-3 activity was measured by AMC liberation from Ac-DEVD-AMC substrate at 380/460 nm using a plate reader (DTX-880 Multimode Detector, Beckman Coulter). Relative fluorescence of substrate control was subtracted as background emission. Free AMC (Enzo Life Sciences) was used to make a standard curve and caspase-3 activity was calculates as

$$\text{Caspase-3 activity (nmol AMC/min/mL)}=\frac{nmol\ AMC}{t\times v}.$$

In our assay, *v*-volume of sample was 0.2 mL and *t*-reaction time was 60 min.

Supplementary figures

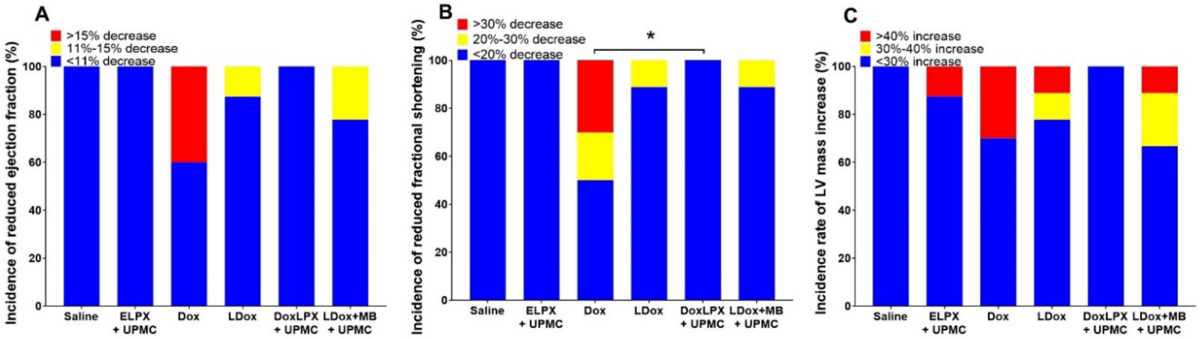

Figure S1. Incidence of change in cardiac function. The percentage of the animals showing different levels of change (*n*=8-9) in (A) ejection fraction; (B) fractional shortening; and (C) LV mass index on Day 21 vs. Day 0. **p*<0.05, Fisher's exact test.

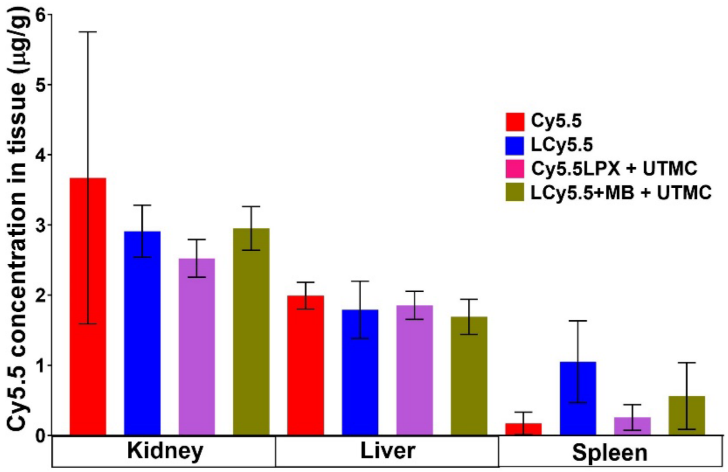

Figure S2. **Quantitative biodistribution of Cy5.5 concentration** in kidney, liver, and spleen in tumor-bearing mice 3.5 h post *i.v.* injection of free Cy5.5, LCy5.5, LCy5.5+MB + UTMC, and Cy5.5 LPX + UTMC (*n*=3).
